# Progress in Scientific and Artistic Dentistry

**Published:** 1884-09

**Authors:** E. Parsons

**Affiliations:** SAVANNAH, GA.


ARTICLE IV.

### PROGRESS IN SCIENTIFIC AND ARTISTIC DENTISTRY.

BY DR. E. PARSONS, SAVANNAH, GA.

*Gentlemen:*—The subject I have chosen for your consideration at this, our annual meeting, is large and broad enough to fill a large volume, and you will please excuse me if I only present an outline of it with incidental remarks.

This subject might be properly treated historically, and thus elucidate its progress. But what we most need now, is the best means for perfecting ourselves in our chosen profession, and thus enlarge our spere of usefulness. The age in which we live is a progressive age, full of new inventions, and in nothing is it more remarkable than in our profession. *Progress, progress,* should be our watchword, and for this end was this Society formed, using the best means to advance both our own and the public good. There is inherent in every human mind a desire to know something that it does not know, and the stronger this desire is, the greater will be the amount of knowledge acquired (opportunities, permitting,) in either an office, profession or calling.

Beyond a doubt, a zeal not according to knowledge has been a fruitful source of error in all ages. Let it not be so with us; science has to do with both apparent, and real truths; the sun appears to move around the earth, but by study, observation and experiment, we are able to demonstrate the contrary, which is the real or scientific truth. The fallacy of the senses often betray us in our search after truth.

It is well known that reflex action will produce a great disturbance in the parts more or less distant from the cause. To illustrate: Many years ago, I had an acute pain in my lower front incisors; I examined them carefully and found them perfect, but on further examination I found the interior third molar decayed at the margin of the gum; it had never given me the slightest indication of the disintegration going on. I had it extracted, and the pain in the incisors was immediately relieved. A large portion of those that suffer from facial neuralgia is caused by defective teeth, You know that any treatment in such cases is useless, unless the cause is removed.

It is only by comparison that we can realize the wonderful progress that has been made in the last fifty years in both the arts and sciences. Fifty years ago the general status of our profession was like a child beginning to walk; I know of but three books avalable then designed to assist the student of dentistry; the authors were, Bell of London. Fitch, of Philadelphia, and Snell, of the same place. Not a single periodical known, devoted to dental information. The teeth then used to supply the lost organs, were mostly human teeth, or carved out of the tusks of the Hippopotamus. About this time, Stocton introduced his mineral teeth; they were so conspiciously different in appearance from the natural ones, that many preferred human teeth.

We took our moulds to a brass foundry and had brass castings to stamp up our base plates. A little later blocks were carved out to suit each case, a sample block I present for your inspection. In 1835, Dr. S. Spooner's work, "Guide to Sound Teeth," appeared, and it was made known the efficacy of arsenic, by which means the nerve of a tooth could be devitalized in a few hours, if properly applied.

In 1843, Godard's Work on the Teeth, with fifty plates illustrating its contents, appeared, and is still of great value to the dental student of anatomy. I need not mention our colleges, for you all know what they have done to advance the cause of dental education.

We now have books and periodicals in abundance, which will compare favorable with those of any other profession, and all may keep themselves posted up if they will.

Progress in mechanical dentistry is as marvelous as in any other department of human industry. The great advance in the manufacture of artificial teeth, from a rude beginning, is now so perfect that, in appearance, they are often mistaken for the natural organs. You can get a set of fourteen for the same price we had to pay for a single human tooth fifty years ago; the introduction of rubber, celluloid, and fusible metal, has added tenfold to the demand for them. The improvements in instruments, and the various modern appliances for executing difficult operations, are beyond all praise.

The scientist is not satisfied until he has ascertained the true cause of the effects that comes within the sphere of his observations; to do this he must diligently study the laws that govern both mind and matter. To illustrate: Take any simple substance, we find the quality of the mass the same as the particle. Take gold; its quality all recognize; the union of its particles forms a large body, and is worked into various forms of use and ornament, and is the standard of all values. The particles are held together by a universal law, and this law we call cohesive attraction. The effect is patent to all, but science alone reveals the cause. The true cause is an eternal active force, operating in both mind and matter; simple as it may seem, this law is like to like, varied only in recipient forms; suspend this law, and we should not be here, everything would be chaos. Gold, by proper manipulation, is brought into a state, when cold, on which this law has increased force; thus we have cohesive gold, creating a new era in the art of filling teeth.

We cannot scientifically treat the various diseases which belong to our specialty, without a knowledge of chemistry physiology, pathology, and therapeutics. There are many carious teeth, if filled without proper treatment, are sure to give trouble afterwards. The preparation for filling a tooth requires scientific knowledge; unless we possess a good degree of mechanical skill also, we cannot do the work in the best manner—science alone, or art alone, cannot produce a well educated dentist. The mechanics may construct fine specimens of art, his materials are void of life, we have to deal with living, and often exceedingly sensitive, tissue; hence a knowledge of the above-named sciences are of great importance to us. Add to these animal magnetism, electricity, and cohesive attraction and motion, and a careful study of the varied phenomena and causative principles of these will add greatly to our ability, both professionally and socially; all cultivated minds respect intellectual acquirements, and elevate the possessor far above the ignorant pretender. Our colleges lay only a good foundation for dental education; after leaving college, the first thing to do is to select a field of labor in which to build up a good reputation, without which no one can succeed in his chosen profession.

A mind well stored with useful knowledge, combined with faithful service, punctuality in business, and a gentlemanly department, will always command the respect of good society.

Again, there is no cohesion between any metallic filling and dentos; the cavity must be formed in strict agreement with mechanical laws or the filling will not serve the purpose intended, and surely disappoint both patient and dentist. Success in this department depends on a practical knowledge of both science and art. Some of our mineral plastic materials cohere and may be held in position in almost any formed cavity, and are highly useful in many cases. The misfortune is, the composition has not yet been discovered that, in appearance and durability, is all our patients desire. Many chemists are now hard at work experimenting with various materials to produce a cement that will chrystalize, resemble the enamel of a tooth, and be as durable as gold. Experiments in this direction are yet in their infancy, and chemical sciences in its future developments may produce this desired result. When we reflect on the inventive genius of the age, we need not be surprised at anything new not heretofore known.

True science is a knowledge of causative principles on any subject the human mind is capable of contemplating. If we have a good knowledge of therapeutics and make a correct diagnosis, we can readily apply an efficient remedy in any case we are called on to treat. The man of science takes into due consideration the varied idiosyncracies or pre-disposing cause of disease ; hence, we need a variety of remedial agents. Every mistake made is like a black spot on a white garment. The uneducated dentist works by guess, and makes many mistakes, often doing more harm than good, and this is our strongest argument in favor of a thorough dental education ; to know when and how to treat all diseases in the oral cavity of all who practice our specialty. Brass, with the ignorant, may pass for gold ; but if we educate the people, the counterfeit will be protected, and such a thing as an ignorant dentist will soon be amongst the things of the past. Whatever our attainments may be, carelessness in manipulation will often seriously damage an otherwise well earned good reputation.

In the winter of 1842, a gentlemen of generally acknowledge ability, visited Savannah, as he had done three or four winters previous, to practice the then known art of dentistry, which consisted almost entirely of filling and cleaning teeth, and substituting artificial ones for the lost organs ; but the very first week after his arrival, in filling a molar tooth he let his instrument slip ; it passed through the cheek and made a very painful wound. He had for a patient one of our most respected ladies. The news spread rapidly, and his intended visit was shortened, as his former patrons failed to call on him. He remained in all but two weeks ; and did not make his expenses ; his patrons preferring to trust the new dentist. His loss was my gain. I mention this incident as a cautain to all. Our patrons will not excuse carelessness.

The subject of this paper requires a brief notice of amalgam, which is now so extensively used in filling teeth. If my memory serves me right, about the year 1833, the Crawcours came to this country to introduce what they named the Royal Succedmeum, which was simply the filling of siver coin made plastic by the addition of murcury; by means of the press, and a bold assertion that it was a better preservative, and as durable as gold, they attracted public attention to this wonderful new discovery. They were for a short time liberally patronized, filling all decayed teeth in the most imperical manner. Those who submitted to their operations soon became disgusted with the horrid discolorations of their teeth, and had the filling removed—everyone condemned it.

The Crawcours sold their secret and left the country. It was some time after, Dr. Towsend, of Philadelphia, added tin, and called it amalgam, advocated its use for filling some badly decayed teeth, asserting that he could save, by its use, some that he could not save with gold. This caused scientific experiments, which have been going on from that day to this, prompted by a desire to produce a compound plastic metal that in the process of hardening would neither shrink nor smell, resist oxidation and be as easily kept clean as the tooth itself. You all know how near this desired end has been attained.

In all compound metallic bodies, each metal retains its identity, and although it may appear to be lost in the mass, the chemist can separate them, and free either of them from all other substances. The causative principle of cohesion, by which the particles of a simple substance are united, and made a large body, if true, by what law can a compound body be formed? Science alone can reveal the mysteries of this problem. What is known as matter in general we divide into four classes, namely; gases, liquids, minerals, and metals. Each class, viewed in itself, is distinctly different, and, as you all know, almost any kind of a compound can be effected with them by proper manipulation.

All things in the created universe, whether it be atoms or worlds, are created for uses. The union of the supreme, and ever active, law of use, conjoined with like to like, are the true causative principles by which means every variety of form ever has been, or ever will be, effected; they operate on the world of matter, as will and understanding operate on the mind. Suspend either of these laws and there would be an end to progress, procreation would cease, there could be no development of anything new, and science and art would be known no more. And now, allow me to say, our proceedings are published in our periodicals, and meet only the eye of the dentist; can we not have at least a synopsis published in a daily paper in Atlanta, Augusta, Columbus, Macon and Savannah? do this, and the public will better understand and appreciate our efforts to rid the State of ignorant pretenders, which, all must acknowledge, is for the public good. We cannot possibly elevate the standard of our profession in this State, as it should be, without the co-operation of its honorable and intelligent citizens.

Progress in science and arts is accelerated ten fold by associated effort; this is as true in our profession as any other; he who seeks to obtain honors for himself alone, does violence to our common humanity, and is an effigy of selfishness, supposing that he knows all that is worth knowing. The true scientist is ever on the alert to increase his knowledge and rationally understand causative principles; he has no fear of exhausting the storehouse of knowledge; every new truth reveals new fields to be explored, and he delights in communicating the same to others, though the infinitely large and the infinitely small will ever remain incomprehensible to the finite mind; yet the telescope has made the astronomer acquainted with the vastness, numberless and oder of the heavenly bodies, and the microscoped has brought into view the wonderful organization of animated nature, and is of vast importance to us who have to treat the various diseases of the oral cavity. Histological science would be of little value to us, if this useful instrument had never been invented. But now we are able to penetrate deep into the heretofore mysterious formation of animal tissue. With this, and the spectroscope, many valuable discoveries have been made in the obnormal conditions of the blood, especially when a person is brought into a state of insensibility by the use of anæsthetic agents. This Society has done much in elevating the standard of dentistry in this State, but vastly more would be accomplished if all honorable members of our profession would make greater exertions to meet with us; no great achievement has ever been made in science and art, without individual sacrifices of both time and money. The best dentist in this State will surely learn something to his advantage by meeting with us, that would far outweigh his expenses.

Any petty jealousies that sometimes exist towards a brother dentist should be buried out of sight, and make the good of all, our good, which is the most exalted state of true manhood. Every graduate of a dental college should meet with us, and help us in our effort to banish quackery from the State. I ask them to consider what this Society has done for their protection? If the self-sacrificing enterprise of a few dentists to organize a State Society, had not been made, the State would be even now over-run with ignorant pretenders, which would be a disgrace to the very name of dentist. In conclusion, I present the following aphorisms, which will benefit all who heed them:

1st. There can be no progress in scientific and artistic dentistry without study, observation and experiment.

2nd. An aversion to any kind of labor is a double tax on the nervous system of the performer, and the only possible satisfaction is the remuneration received for it.

3rd. Without a love for a chosen profession, opportunities for improvement are neglected, no progress made, what is done, is from necessity, and not from any pleasure therein.

4th. A laudable ambition to excel in what we undertake to do, is the high-road to eminence, and always deserves success.

5th. To make this life a happy one, the golden rule should be our guide in all we do.

6th. Dissipation and debts are the two greatest enemies to human happiness; always avoid them.—*Dental Luminany*.

